# JAK-STAT Signaling: Mechanisms and Therapeutics in Stem Cell Biology

**DOI:** 10.3390/ijms27209052

**Published:** 2026-10-13

**Authors:** Willis X. Li

**Affiliations:** Department of Medicine, University of California, San Diego, CA 92093, USA; wxli@health.ucsd.edu

**Keywords:** JAK-STAT, stem cells, cancer stem cells, niche, metabolism, epigenetics, regenerative medicine, targeted therapy

## Abstract

The Janus kinase–signal transducer and activator of transcription (JAK-STAT) pathway is a conserved signaling system that links extracellular cytokine signals to transcriptional regulation of genes controlling cell proliferation, differentiation, and survival. Increasing evidence demonstrates that this pathway plays central roles in stem cell biology across diverse systems, including pluripotent stem cells, adult tissue stem cells, and cancer stem cells. In pluripotent stem cells, STAT3 signaling downstream of leukemia inhibitory factor (LIF) supports self-renewal and maintenance of the undifferentiated state, whereas in adult stem cells, JAK-STAT activity regulates the balance between quiescence, proliferation, and lineage commitment. Stem cell niches further modulate pathway activity through spatially and temporally controlled cytokine signals that coordinate regenerative responses with tissue homeostasis. Recent studies have also revealed noncanonical functions of STAT proteins, including mitochondrial STAT3-dependent metabolic regulation and unphosphorylated STAT-mediated chromatin and heterochromatin regulation that influence stem cell identity and function. Dysregulation of the pathway contributes to pathological conditions, including inflammatory disorders and cancers characterized by stem-like cell populations. Therapeutic targeting of JAK-STAT signaling therefore represents an important strategy in both regenerative medicine and oncology. This review summarizes recent advances in understanding how JAK-STAT signaling regulates stem cell biology, highlighting emerging mechanisms of pathway integration, niche regulation, and therapeutic modulation.

## 1. Introduction

Stem cells possess long-term self-renewal capacity and the ability to generate differentiated progeny that maintain tissue homeostasis and support regeneration following injury [1,2]. These properties are regulated by complex signaling networks that integrate extracellular cues with intrinsic transcriptional and metabolic programs. Among these, the Janus kinase–signal transducer and activator of transcription (JAK-STAT) pathway represents a direct and evolutionarily conserved mechanism for translating extracellular signals into transcriptional responses [3,4,5,6].

Originally characterized as a rapid cascade linking cytokine receptor activation to nuclear gene expression, JAK-STAT signaling begins with cytokine binding, which activates receptor-associated JAK kinases [7,8,9,10]. Activated JAKs phosphorylate STAT transcription factors, prompting dimerization, nuclear translocation, and regulation of genes controlling proliferation, differentiation, and survival. Its relatively simple architecture allows cells to respond efficiently to environmental changes [3,4,5,6,11].

In stem cell systems, JAK-STAT signaling is essential for maintaining identity and balancing self-renewal with differentiation. STAT3 activation is critical for pluripotency in mouse embryonic stem cells via LIF-mediated signaling [12,13,14], and the pathway also regulates adult stem cell populations, including hematopoietic, epithelial, and neural systems, where cytokine-mediated signaling influences proliferation, quiescence, and lineage commitment. In *Drosophila*, JAK-STAT signaling governs germline stem cell maintenance within their niche, highlighting evolutionary conservation and context-dependent regulation [15,16,17]. The *Drosophila* system has been particularly powerful for dissecting how JAK-STAT signaling operates within intact stem cell niches, revealing principles of spatial signaling gradients, stem cell competition, and non-autonomous regulation that are conserved in mammalian systems [18,19,20,21,22,23,24,25,26].

Stem cell behavior is strongly influenced by niche interactions, which produce cytokines and growth factors regulating JAK-STAT activity. Through these microenvironmental signals, the pathway integrates immune responses, tissue injury cues, and metabolic states to coordinate regeneration with tissue homeostasis [21,27,28,29,30,31]. In multiple systems, including hematopoietic and epithelial tissues, niche-derived cytokines such as IL-6 family members and interferons dynamically modulate STAT activation in response to stress and inflammation [32,33]. STAT proteins further interact with epigenetic regulators and metabolic pathways to shape gene expression programs that determine stem cell fate [34,35,36,37]. In addition, evidence from *Drosophila* and mammalian systems demonstrates that STAT contributes to chromatin organization and heterochromatin stability, highlighting conserved noncanonical roles in epigenetic regulation [38,39,40,41].

Dysregulation of JAK-STAT signaling has pathological consequences. Persistent STAT activation is frequently observed in inflammatory diseases and cancers with stem-like tumor cell populations [42,43]. Aberrant activation drives uncontrolled proliferation, survival signaling, and altered differentiation, contributing to tumor progression and therapeutic resistance [44,45,46]. In hematologic malignancies and solid tumors, constitutive activation of STAT3 and STAT5 supports cancer stem cell maintenance, immune evasion, and resistance to therapy [4,47,48,49,50,51]. Chronic cytokine signaling, particularly through IL-6–JAK-STAT3 pathways, further reinforces oncogenic feedback loops within the tumor microenvironment [52,53,54,55,56,57]. These observations have made JAK-STAT a major pharmacological target in oncology and immunology, leading to the development of JAK inhibitors and emerging STAT-targeted therapies [58,59,60,61,62].

In this review, we summarize recent advances in understanding JAK-STAT signaling in stem cell biology, emphasizing mechanisms by which cytokine signaling regulates pluripotent and adult stem cells, the influence of stem cell niches, integration with metabolic and epigenetic networks, and implications of pathway dysregulation in cancer stem cells and therapeutic development.

## 2. Overview of the JAK-STAT Signaling Pathway

The JAK-STAT pathway is a conserved mechanism transmitting extracellular cytokine signals directly to the nucleus, enabling rapid transcriptional responses. It consists of cytokine receptors, Janus kinases (JAKs), and signal transducers and activators of transcription (STATs). Mammals express four JAKs (JAK1, JAK2, JAK3, and TYK2) and seven STAT family members (STAT1, STAT2, STAT3, STAT4, STAT5A, STAT5B, and STAT6), forming overlapping signaling networks [3,5,63].

Cytokine binding induces receptor dimerization, bringing receptor-associated JAKs into proximity for trans-phosphorylation. JAKs then phosphorylate receptor tyrosine residues, creating docking sites for STATs. STATs are phosphorylated, dimerize, and translocate to the nucleus to regulate genes controlling proliferation, differentiation, and survival [8,64,65]. Serine phosphorylation and other modifications further refine STAT target specificity [66].

Regulation of JAK-STAT signaling involves multiple mechanisms: suppressor of cytokine signaling (SOCS) proteins, protein inhibitors of activated STATs (PIAS), and tyrosine phosphatases like SHP1/2 attenuate signaling, while receptor internalization and degradation ensure transient pathway activity [67,68,69].

Beyond canonical phosphorylation-dependent transcription, STATs have noncanonical functions. STAT3 can localize to mitochondria and the endoplasmic reticulum (ER) to modulate metabolism [70,71], and unphosphorylated STATs regulate chromatin and gene expression independently of cytokine signaling [38,72]. Research studies reveal that Stat92E maintains heterochromatin structure through HP1, linking JAK signaling to epigenetic regulation and genome stability [39,73]. Recent work shows that STAT’s phosphorylation state determines its partitioning between transcriptional and chromatin-associated functions, supporting germline stem cell maintenance and epigenetic integrity [40].

**Figure 1:**
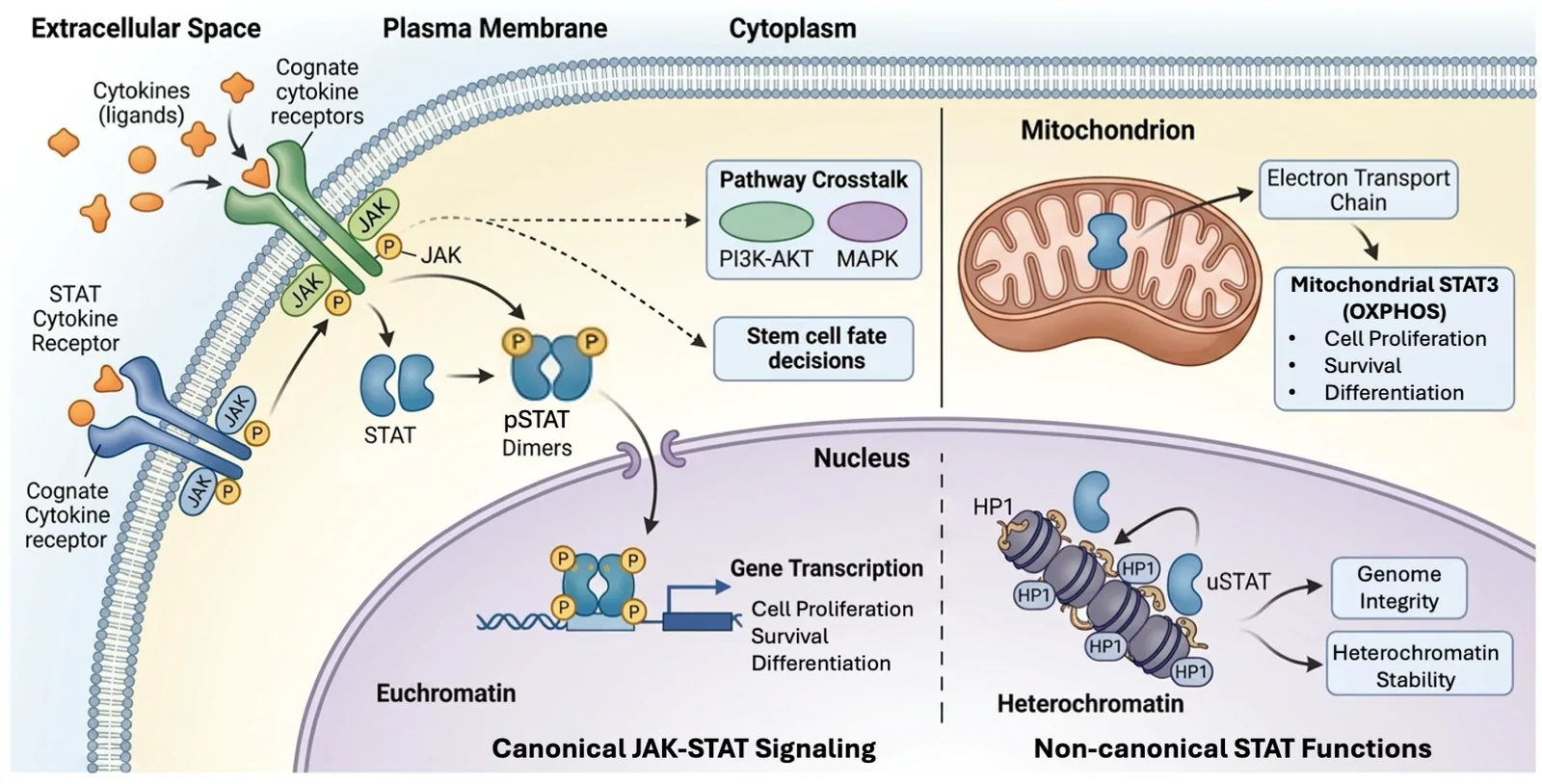
Canonical and noncanonical JAK-STAT signaling mechanisms. Schematic representation of the JAK-STAT signaling pathway. Binding of cytokines to their cognate receptors induces receptor dimerization and activation of receptor-associated Janus kinases (JAKs). Activated JAKs phosphorylate intracellular receptor domains and STAT transcription factors, promoting STAT dimerization and nuclear translocation. STAT dimers regulate transcription of genes involved in cell proliferation, survival, and differentiation. In addition to canonical transcriptional signaling, noncanonical STAT functions have been reported, including mitochondrial localization of STAT3, which modulates oxidative phosphorylation and electron transport chain activity, and heterochromatin-associated unphosphorylated STAT (uSTAT), which maintains heterochromatin stability and genome integrity. STAT signaling also intersects with other pathways (e.g., PI3K–AKT, MAPK) to integrate diverse extracellular cues and fine-tune stem cell fate decisions. Arrows indicate the direction of molecule movement.

This dual functionality includes rapid transcriptional responses and chromatin regulation. Such integration positions JAK-STAT as a central coordinator of stem cell fate, linking extracellular signals to long-term maintenance of cellular identity and genome stability, with implications for both stem cell biology and cancer (Figure 1) [38,72,74].

## 3. JAK-STAT Signaling in Pluripotent Stem Cells

Pluripotent stem cells (PSCs) generate all embryonic lineages and maintain self-renewal through a transcriptional network stabilized by JAK-STAT signaling, particularly STAT3 in mouse ESCs (mESCs) [12,75,76,77]. LIF activates the LIFR/gp130 receptor, inducing JAK-mediated phosphorylation of STAT3, which is both necessary and sufficient for self-renewal [78,79]. Experimental STAT3 activation alone preserves pluripotency, while inhibition triggers differentiation. STAT3 also reinforces the naïve pluripotency network by promoting Klf4, Tfcp2l1, and other key transcription factors [14,35,80,81].

STAT3 integrates with the core pluripotency triad (OCT4, SOX2, and NANOG), co-occupying enhancer regions to coordinate transcription [82]. It buffers against differentiation cues, particularly FGF–ERK signals, and, in combination with dual MEK/GSK3 inhibition (“2i”), locks ESCs into a naïve ground state resembling the preimplantation epiblast [83,84]. Naïve and primed PSC states differ in STAT3 dependency: naïve mESCs rely heavily on LIF–STAT3, while primed epiblast stem cells depend on FGF/Activin and respond less to STAT3 [85]. STAT3 activation can revert primed cells toward naïve-like states, defining a functional boundary between pluripotent states [81].

In human PSCs, conventional ESCs and iPSCs are largely in a primed state, relying on FGF2 and Activin/Nodal rather than LIF [86]. Naïve-like human PSCs can acquire or regain STAT3 responsiveness under defined culture conditions, highlighting species-specific differences [81]. During reprogramming to iPSCs, STAT3 enhances efficiency and supports acquisition of stable pluripotency, cooperating with KLF4 to reinforce transcriptional networks [87]. Nevertheless, excessive STAT3 activity is deleterious; SOCS proteins and phosphatases constrain signaling, preventing aberrant proliferation and oncogenic transformation [88].

**Figure 2:**
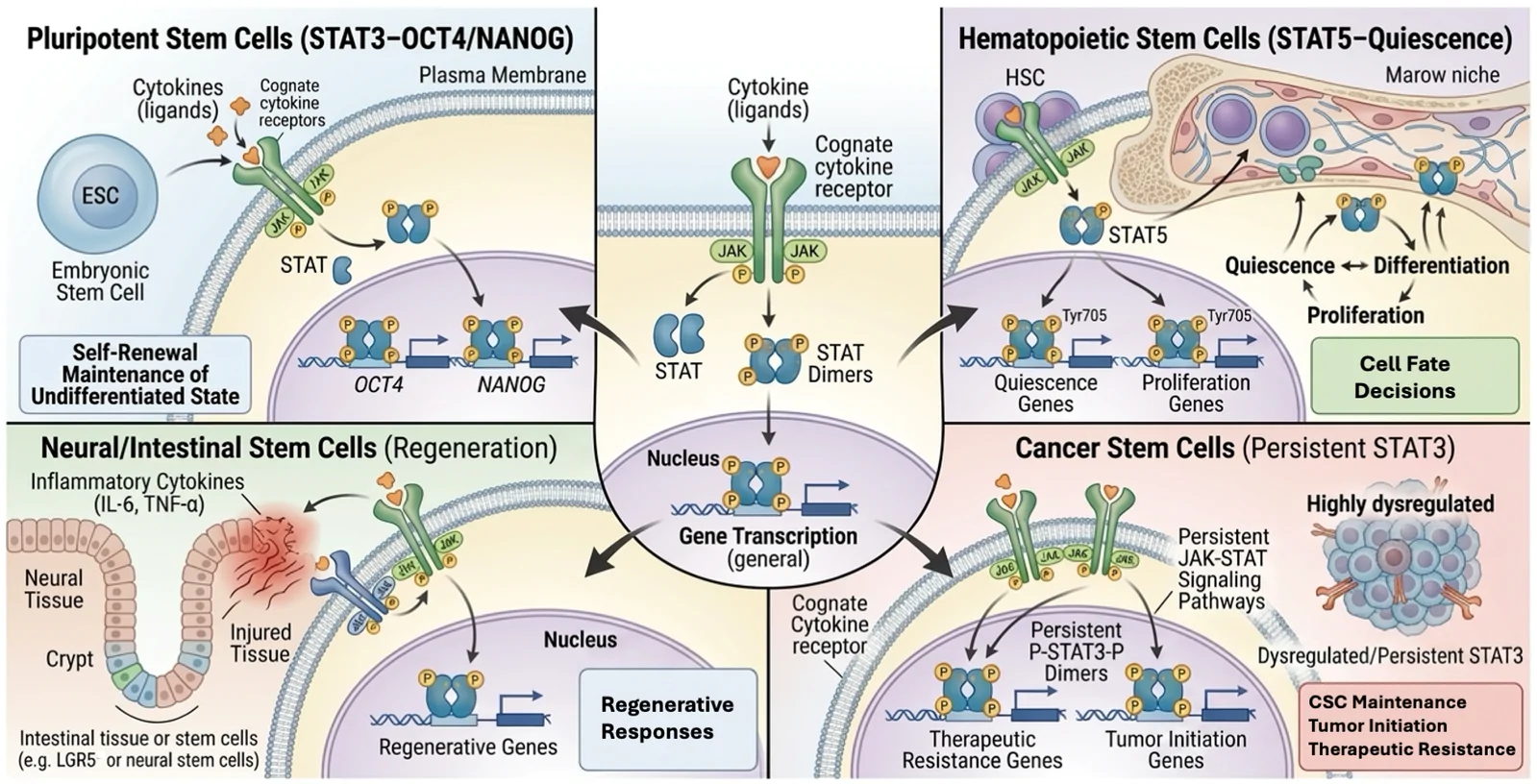
Context-dependent roles of JAK-STAT signaling in stem cell regulation. JAK-STAT signaling regulates multiple aspects of stem cell biology across different cellular contexts. In pluripotent stem cells, STAT3 signaling promotes maintenance of the undifferentiated state and supports self-renewal. In adult stem cells such as hematopoietic stem cells, cytokine-mediated STAT activation regulates transitions between quiescence, proliferation, and differentiation. In tissue-resident stem cells, inflammatory cytokines can activate STAT pathways to stimulate regenerative responses following injury. Dysregulated STAT signaling can also contribute to the maintenance of cancer stem cells, promoting tumor initiation and therapeutic resistance. Arrows indicate the direction of molecule movement.

In summary, STAT3 functions as a master regulator of naïve pluripotency, particularly in mouse ESCs, by integrating extracellular cues with the core transcriptional network and epigenetic machinery. Its context-dependent activity across species has important implications for maintaining and directing pluripotent stem cells for regenerative applications. Therapeutic strategies that modulate STAT3 activity may therefore need to balance maintenance of pluripotency with controlled differentiation, an issue relevant to the clinical development of stem-cell-based therapies (Figure 2).

## 4. JAK-STAT Signaling in Adult Stem Cell Regulation

Adult stem cells maintain tissue homeostasis by balancing long-term self-renewal with production of differentiated progeny. In many tissues, cytokine-mediated activation of JAK-STAT signaling plays a central role in regulating these processes [21,27,89]. Hematopoietic stem cells (HSCs) represent one of the best-characterized systems in which JAK-STAT signaling controls stem cell behavior, influencing quiescence, proliferation, and lineage specification [37,90].

HSCs sustain lifelong blood production through tightly regulated self-renewal and multilineage differentiation [91,92]. Cytokines, including thrombopoietin, erythropoietin, interleukins, and interferons, activate JAK kinases and downstream STAT transcription factors in hematopoietic stem and progenitor cells [93,94]. STAT5, in particular, is critical for hematopoietic stem cell expansion and survival [95]. Loss of STAT5 impairs erythroid and lymphoid development, whereas constitutive activation enhances stem cell self-renewal and competitive repopulating capacity [96]. However, sustained hyperactivation skews lineage output and predisposes cells to myeloproliferative disease [97], illustrating the importance of precise regulation of signaling intensity. Tyrosine-unphosphorylated STAT5 (uSTAT5) is also important in maintaining HSCs [37], possibly involving heterochromatin regulation [40,73,98]. The molecular mechanisms underlying these dosage-dependent effects of JAK-STAT signaling in hematopoietic stem and progenitor cells have been extensively reviewed, highlighting how pathway activity levels dictate the balance between self-renewal, lineage commitment, and leukemic transformation [90,95].

Adult HSCs normally reside in a quiescent state that protects genomic integrity and preserves long-term regenerative potential [99]. JAK-STAT signaling participates in transitions between quiescence and proliferative activation in response to physiological demands [37]. Type I interferons activate STAT1 signaling and drive dormant HSCs into the cell cycle during infection or stress responses [100]. While transient activation can enhance regenerative output, chronic stimulation leads to stem cell exhaustion, demonstrating that temporal regulation of cytokine signaling is essential for maintaining stem cell homeostasis [101].

The bone marrow microenvironment provides critical regulatory signals that shape JAK-STAT activity in hematopoietic stem cells [90]. The niche consists of mesenchymal stromal cells, endothelial cells, osteoblasts, and neural elements that produce cytokines and chemokines influencing stem cell behavior [102]. CXCL12-producing stromal cells play a key role in maintaining HSC quiescence and retention within perivascular regions of the marrow [103]. Spatial gradients of cytokines within the niche create localized zones of STAT activation, contributing to heterogeneity in stem cell behavior across the hematopoietic hierarchy [104].

**Figure 3:**
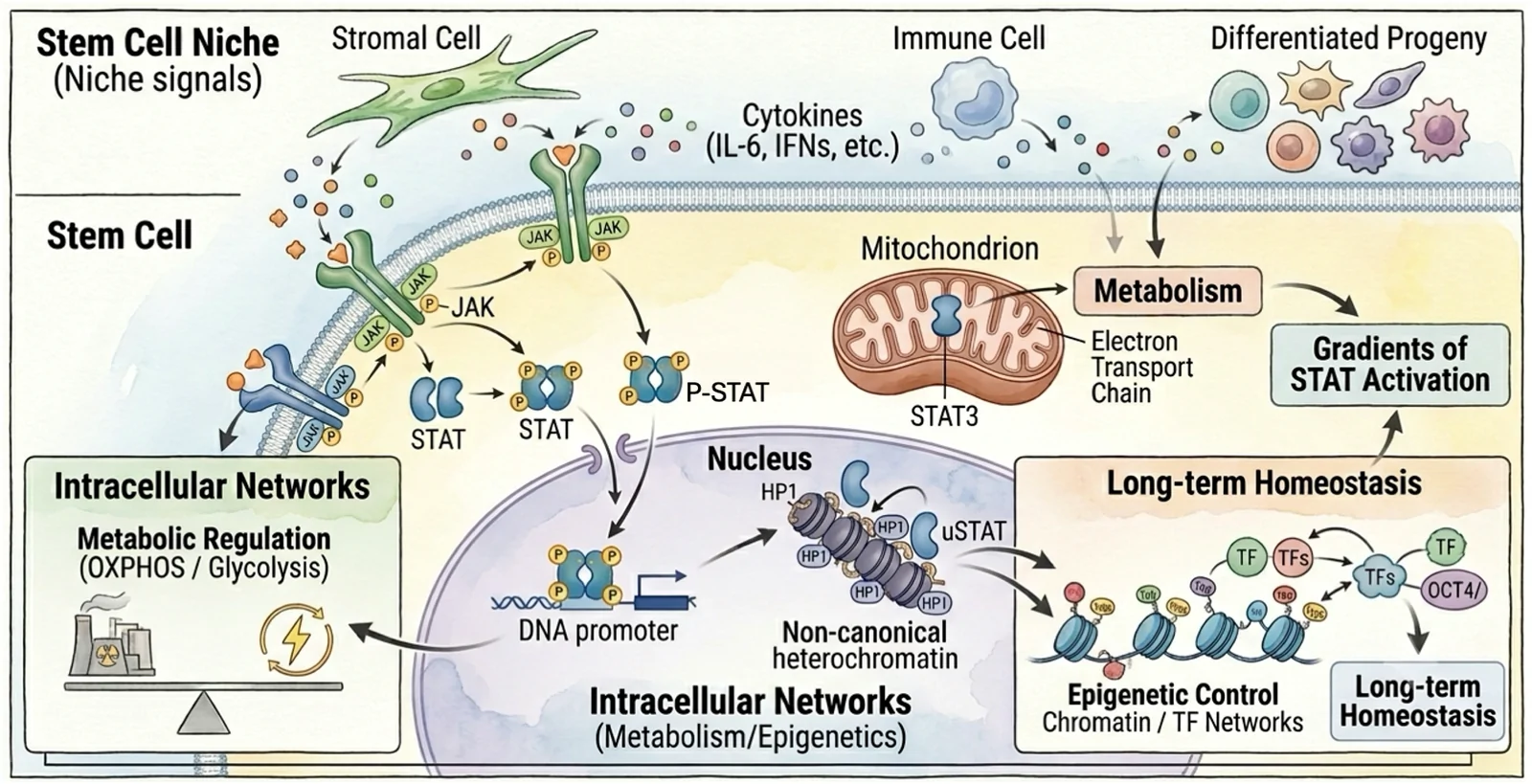
Integration of JAK-STAT signaling with stem cell niches and regulatory networks. Stem cell behavior is influenced by signals from the surrounding microenvironment. Cytokines produced by stromal cells, immune cells, and differentiated progeny activate JAK-STAT signaling in nearby stem cells. This pathway integrates extracellular cues with intracellular regulatory networks, including metabolic pathways and epigenetic regulators. Spatial and temporal control of cytokine signaling within the niche generates gradients of STAT activation that influence stem cell fate decisions, allowing tissues to balance regenerative responses with long-term homeostasis. Arrows indicate the direction of molecule movement.

Beyond hematopoiesis, JAK-STAT signaling regulates tissue-resident stem cells in multiple organs. In epithelial tissues such as the intestine and skin, inflammatory cytokines activate STAT3 signaling to stimulate regenerative proliferation following injury [105,106]. Similar mechanisms operate in neural stem cells, where cytokine-mediated STAT activation influences lineage specification and tissue repair [107]. Together, these findings indicate that JAK-STAT activity is highly context-dependent, with its effects on stem cell maintenance and regeneration determined by tissue type, cytokine environment, and signaling intensity (Figure 3) [65,108].

## 5. Stem Cell Niches and Microenvironmental Control

Stem cell behavior is profoundly influenced by the microenvironment or niche in which stem cells reside. The niche provides structural support and delivers biochemical signals that regulate stem cell quiescence, proliferation, differentiation, and survival. JAK-STAT signaling frequently acts as a key mediator of these niche-derived signals, integrating cytokine inputs with intrinsic transcriptional programs that determine stem cell fate. Cytokines produced by stromal cells, immune cells, and differentiated progeny can activate JAK kinases and STAT transcription factors in neighboring stem cells, thereby coordinating tissue maintenance with environmental conditions [21,109,110].

Paracrine signaling is a major mechanism through which niches regulate stem cells. In many tissues, cytokines secreted by stromal or immune cells activate STAT signaling pathways in nearby stem cells. For example, IL-6 family cytokines activate STAT3 to stimulate regenerative proliferation during tissue repair. Similar mechanisms operate in intestinal, neural, and epithelial tissues, where inflammatory signals transiently enhance stem cell proliferation to restore tissue integrity following injury [111,112,113]. The *Drosophila* midgut provides a powerful model for this regenerative mechanism, where damage-induced enterocyte apoptosis triggers production of the cytokine Unpaired (Upd), which activates JAK-STAT signaling in adjacent intestinal stem cells to drive their proliferation and subsequent differentiation, thereby restoring epithelial integrity [114]. Autocrine signaling also contributes to JAK-STAT regulation, as stem cells themselves can produce cytokines that reinforce signaling loops controlling self-renewal and differentiation [27,115].

Insights from *Drosophila melanogaster* have provided a particularly clear mechanistic paradigm for niche-mediated JAK-STAT regulation, which can be compared with related mechanisms identified in mammalian tissues. In the testis niche, specialized hub cells secrete the cytokine Unpaired (Upd), which activates JAK-STAT signaling in adjacent germline stem cells (GSCs) and cyst stem cells, thereby promoting self-renewal and preventing differentiation [21,27,116,117]. The restricted production of Upd establishes a short-range signaling gradient, ensuring that only stem cells in direct contact with the niche maintain high STAT activity, while displaced daughter cells experience reduced signaling and initiate differentiation [15,16,21]. This spatially confined cytokine signaling exemplifies how niches impose positional control over stem cell fate decisions. Further work has shown that JAK-STAT signaling also regulates the initial establishment of the male germline stem cell pool during embryogenesis, before the niche is fully formed, indicating additional roles in stem cell specification [118].

Spatial organization within niches further influences JAK-STAT signaling dynamics. Stem cells are often positioned near specialized stromal cells that produce cytokine gradients, generating localized zones of signaling activity [103,119,120,121]. In the bone marrow, for example, perivascular stromal cells produce CXCL12 and other factors that maintain hematopoietic stem cell quiescence and retention within the niche [104,122,123]. Similar spatial arrangements occur in epithelial tissues, where stem cells at the base of regenerative compartments receive signals that differ from those experienced by differentiated progeny [124]. Such spatial heterogeneity creates gradients of STAT activation that contribute to differential gene expression across the stem cell hierarchy, a principle conserved from *Drosophila* niches to mammalian tissues [115,125]. A particularly striking example of JAK-STAT-mediated niche regulation comes from studies of stem cell competition in the *Drosophila* testis, where differences in STAT activity determine which germline stem cells are retained within the niche and which are displaced, ensuring optimal stem cell pool quality [18,19]. In the *Drosophila* intestine, JAK-STAT signaling does not operate in isolation but cooperates extensively with EGFR, Hippo, and Wingless pathways to coordinate stem cell proliferation and lineage decisions, illustrating how multiple niche-derived signals are integrated at the stem cell level [126,127].

Although *Drosophila* studies have provided important mechanistic insights into how localized JAK-STAT signaling regulates stem cell–niche interactions, the extent to which these mechanisms are conserved in human tissues requires further investigation. In particular, the principles of short-range cytokine gradients, differential STAT activation according to stem cell position, and niche-dependent stem cell competition should be tested in human primary stem and progenitor cells and in physiologically relevant tissue models. Human intestinal and other epithelial organoids, bone marrow stem/progenitor cell cultures, and co-culture systems incorporating stromal or immune cells could help determine whether spatially restricted cytokine signaling similarly controls human stem cell proliferation, quiescence, and differentiation. Single-cell and spatial transcriptomic approaches, together with multiplexed imaging and functional perturbation of JAK-STAT components, may further define the cellular sources, spatial distribution, and temporal dynamics of pathway activity in human tissues. Such studies will help distinguish conserved mechanisms from species-specific features and establish which components of niche-mediated JAK-STAT signaling may have translational or therapeutic relevance.

Temporal regulation of cytokine signaling is also essential for maintaining stem cell homeostasis. Acute activation of JAK-STAT signaling during injury or infection can stimulate regenerative proliferation, whereas chronic activation may lead to stem cell exhaustion or pathological expansion. Consequently, feedback regulators such as suppressor of cytokine signaling (SOCS) proteins and protein tyrosine phosphatases act to dampen JAK-STAT signaling after activation, preventing sustained pathway activity that could disrupt tissue homeostasis [18,128,129].

**Figure 4:**
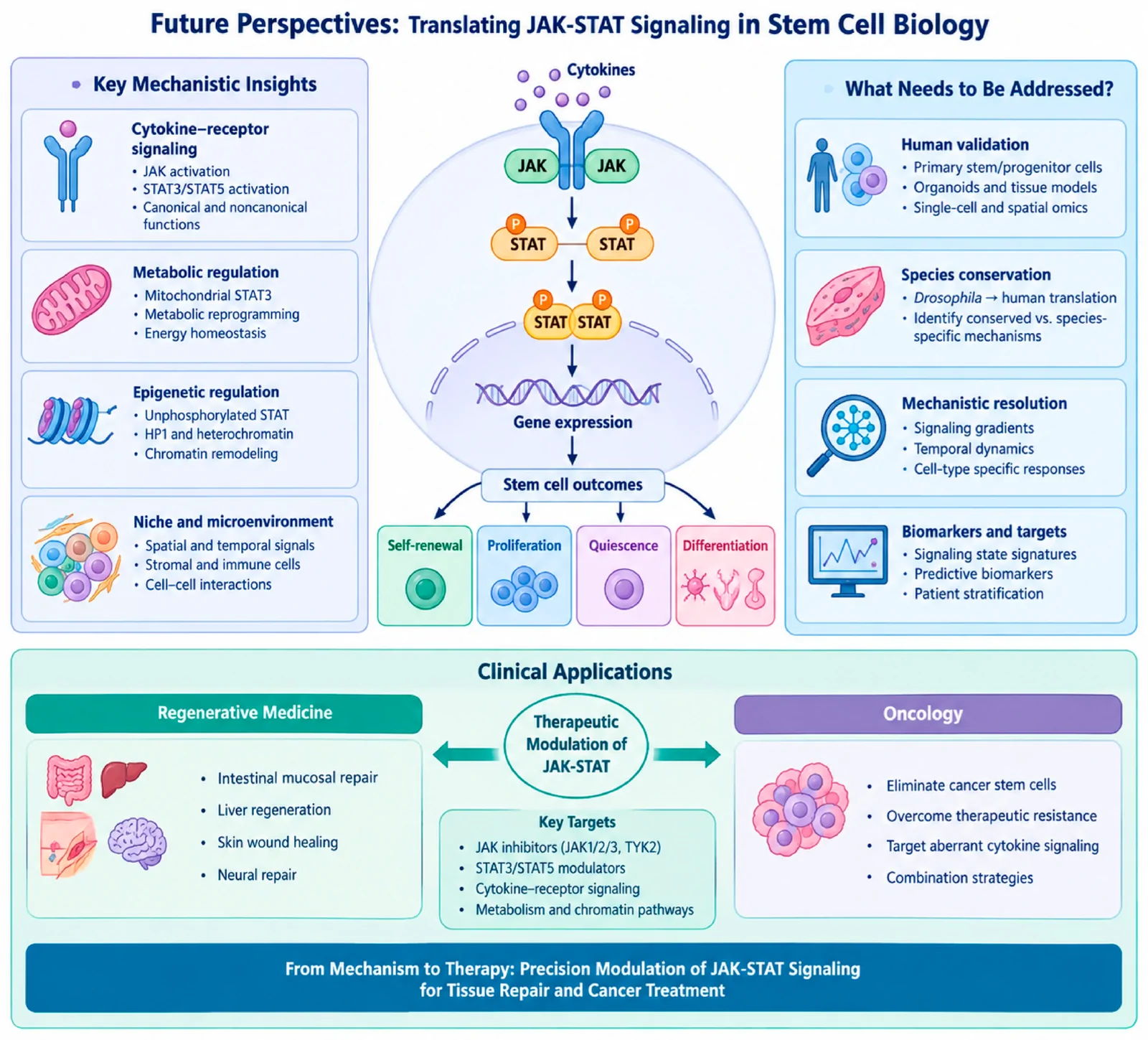
Future perspectives and translational opportunities for JAK-STAT signaling in stem cell biology. The figure summarizes emerging directions for translating mechanistic insights into JAK-STAT signaling into therapeutic applications in regenerative medicine and oncology. Key mechanistic areas include canonical cytokine–receptor–JAK-STAT signaling, noncanonical STAT functions in mitochondrial metabolism and chromatin regulation, and spatial and temporal regulation by stem cell niches and the tissue microenvironment. Major priorities for future research include validation of these mechanisms in human stem and progenitor cells and physiologically relevant organoid or tissue models, determination of conserved versus species-specific mechanisms, characterization of signaling gradients and cell-type-specific responses, and identification of signaling-state biomarkers and therapeutic targets. These advances may support more precise modulation of JAK-STAT signaling through JAK inhibitors, STAT3/STAT5-directed approaches, cytokine–receptor modulation, and metabolic or chromatin-associated pathways for applications in tissue regeneration and cancer treatment. Arrows indicate the direction of molecule movement.

Through these spatial and temporal mechanisms, stem cell niches regulate JAK-STAT signaling to coordinate stem cell maintenance, differentiation, and tissue organization. The *Drosophila* niche provides a particularly clear example of how localized cytokine signaling establishes positional differences in STAT activity, while mammalian studies indicate that related principles operate through stromal, immune, and epithelial signals during tissue homeostasis and injury. Defining how these mechanisms are conserved and functionally regulated in human tissues will be important for translating niche-mediated JAK-STAT biology into therapeutic strategies for tissue repair and disease (Figure 4). The clinical and therapeutic implications of modulating JAK-STAT signaling in regenerative medicine and disease are discussed further in Section 8.

## 6. Metabolic and Epigenetic Integration of Canonical and Noncanonical JAK-STAT Signaling in Stem Cells

Stem cell function is closely linked to cellular metabolism and epigenetic state. Increasing evidence indicates that JAK-STAT signaling integrates extracellular cytokine signals with metabolic and chromatin regulatory networks to coordinate stem cell fate decisions. STAT transcription factors regulate genes involved in mitochondrial activity, glycolysis, and biosynthetic pathways, thereby linking environmental signals to metabolic programs that support stem cell maintenance and proliferation [35,130].

Quiescent stem cells are generally metabolically quiescent, with low oxidative phosphorylation (OXPHOS) activity and dependence on glycolysis. This configuration minimizes reactive oxygen species (ROS) production and helps preserve genomic integrity. Activation of cytokine signaling through JAK-STAT pathways can shift this balance by inducing transcriptional programs that promote mitochondrial function and biosynthetic activity during regenerative proliferation [131,132]. In hematopoietic stem cells, transitions between quiescence and activation involve coordinated changes in metabolic pathways, protein synthesis, and cell-cycle regulators [133].

In addition to transcriptional regulation, STAT proteins can directly influence metabolic processes through noncanonical mechanisms. Mitochondrial localization of STAT3 has been shown to modulate oxidative phosphorylation and electron transport chain activity, demonstrating that STAT proteins can regulate metabolism independently of their nuclear transcriptional functions [134,135]. These mitochondrial STAT3 findings derive in part from broader cellular systems and should therefore be distinguished from direct evidence in bona fide stem cells. These findings, together with broader evidence for unphosphorylated STAT functions, highlight the versatility of JAK-STAT signaling in coordinating cellular responses to environmental cues.

Epigenetic regulation provides another layer of integration between cytokine signaling and stem cell identity. STAT proteins recruit chromatin-modifying enzymes and interact with epigenetic regulators to control accessibility of lineage-specific genes. For example, STAT3 cooperates with pluripotency factors and chromatin remodelers to regulate gene expression programs in stem cells [34,136,137]. Cytokine-driven STAT activation can reshape the epigenetic landscape, enabling dynamic transitions between self-renewal and differentiation states. In parallel, studies in *Drosophila* have demonstrated that STAT directly contributes to heterochromatin organization and genome stability, revealing a conserved noncanonical role in chromatin regulation that extends beyond transcriptional control [39,74,138,139], which is conserved from *Drosophila* to mammals as revealed by the following mammalian studies [73,140]. These noncanonical chromatin functions of STAT are particularly important in the germline stem cell lineage, where STAT’s phosphorylation state determines its partitioning between transcriptional activation and heterochromatin maintenance, thereby coupling cytokine signaling with long-term epigenetic integrity [40,98].

There is an integration of canonical and noncanonical STAT functions in stem cell systems. In *Drosophila* germline stem cells, STAT activity coordinates transcriptional outputs with chromatin organization to maintain stem cell identity and niche interactions [40]. Complementary computational modeling approaches have revealed that differences in JAK-STAT signaling dynamics between somatic and germline stem cells can generate distinct regulatory outcomes, emphasizing how pathway kinetics and network structure contribute to cell-type-specific regulation [128]. These findings highlight the importance of considering both biochemical signaling and systems-level properties of the pathway.

Collectively, these findings indicate that canonical and noncanonical JAK-STAT functions are closely integrated with metabolic and epigenetic regulation in stem cells. Rather than acting solely as a transcriptional relay, STAT signaling can influence mitochondrial metabolism and chromatin organization, providing additional mechanisms through which stem cells respond to physiological and environmental changes.

## 7. JAK-STAT Dysregulation and Cancer Stem Cells

Aberrant activation of JAK-STAT signaling is a hallmark of many cancers and plays a particularly important role in the maintenance of cancer stem cells (CSCs). CSCs represent a subpopulation of tumor cells with self-renewal capacity and the ability to drive tumor growth, metastasis, and therapeutic resistance. Persistent STAT activation, particularly of STAT3 and STAT5, promotes transcriptional programs that support stem-like properties, survival signaling, and immune evasion in malignant cells [47,48].

In hematologic malignancies, constitutive activation of JAK2 or STAT5 can drive uncontrolled proliferation of stem and progenitor cells. Mutations such as JAK2 V617F result in persistent pathway activation and are a defining feature of several myeloproliferative neoplasms. These mutations enhance cytokine signaling sensitivity and promote expansion of malignant stem cell populations [141,142]. Similar mechanisms operate in leukemia, where dysregulated JAK-STAT signaling contributes to leukemic stem cell maintenance, altered self-renewal programs, and resistance to therapy [143,144].

Solid tumors also exploit STAT signaling to maintain cancer stem cell populations, where studies use bulk tumor cells or other malignant cell populations rather than experimentally defined CSCs; these findings provide mechanistic context but do not by themselves establish a CSC-specific function. STAT3 activation promotes epithelial–mesenchymal transition (EMT), enhances tumor-initiating capacity, and regulates genes involved in proliferation, survival, and angiogenesis. Elevated STAT3 activity has been observed in breast, glioblastoma, colorectal, and pancreatic cancers, where it contributes to tumor progression and metastatic potential [4,44,49,145]. In colorectal cancer specifically, persistent JAK-STAT activation promotes cancer stem cell maintenance, drives epithelial-mesenchymal transition, and confers resistance to chemotherapy and targeted agents, making the pathway an attractive therapeutic target in this malignancy [146]. In addition, STAT3 signaling supports the maintenance of CSC phenotypes by regulating core stemness-associated transcription factors such as NANOG, SOX2, and OCT4 [147]. Importantly, STAT3 also modulates the tumor microenvironment by regulating inflammatory cytokines that reinforce oncogenic signaling loops, including IL-6-mediated autocrine and paracrine activation [45,148].

Therapeutic resistance is another key feature associated with JAK-STAT-driven cancer stem cells. Persistent STAT signaling can upregulate anti-apoptotic genes (e.g., BCL-XL, MCL1) and drug efflux transporters, enabling tumor cells to survive chemotherapy and targeted therapies [149,150]. Moreover, JAK-STAT signaling contributes to immune evasion by regulating Programmed Death-Ligand 1 (PD-L1) expression and shaping an immunosuppressive tumor microenvironment [151,152]. As a result, JAK-STAT signaling pathways have become major targets for the development of anti-cancer therapies aimed at eliminating malignant stem cell populations, including JAK inhibitors and STAT3-targeted strategies currently under clinical investigation [153,154].

## 8. Therapeutic Targeting and Clinical Implications in Regenerative Medicine and Oncology

Given its central role in stem cell regulation and oncogenesis, the JAK-STAT pathway has become an important target for therapeutic intervention. Pharmacological inhibitors of JAK kinases have been developed to treat inflammatory disorders, hematologic malignancies, and other diseases characterized by aberrant cytokine signaling (Table 1). Several JAK inhibitors are now approved for clinical use, including ruxolitinib, tofacitinib, and baricitinib, which target specific JAK family members to suppress pathological signaling [155,156,157]. In addition, newer agents such as fedratinib and upadacitinib further expand the therapeutic landscape by offering increased selectivity and alternative safety profiles [158,159].

In hematologic malignancies such as myelofibrosis and polycythemia vera, JAK inhibitors reduce disease symptoms and decrease inflammatory cytokine production by blocking constitutive JAK-STAT activation. These therapies demonstrate the clinical relevance of targeting cytokine signaling pathways in diseases driven by aberrant stem cell expansion [64,160]. However, while JAK inhibitors improve clinical outcomes, they often do not fully eradicate malignant stem cells, highlighting the need for combination strategies targeting downstream or parallel pathways [161]. Emerging evidence indicates that the bone marrow microenvironment plays a critical role in this therapeutic resistance, as different niche compartments can support malignant stem cells carrying the same oncogenic JAK2 mutation, leading to variable responses to JAK inhibitor treatment [162].

Beyond oncology, substantial preclinical evidence supports JAK-STAT signaling as a regulator of tissue regeneration. In skin wound healing, STAT3 activation promotes keratinocyte proliferation, migration, survival, and re-epithelialization, while modulation of STAT3-regulatory pathways can improve healing in experimental chronic and diabetic wounds [163]. In the intestinal epithelium, IL-22–JAK-STAT3 signaling promotes epithelial survival, proliferation, and mucosal restitution following injury [164,165]. Importantly, JAK inhibitors have entered clinical practice for inflammatory bowel diseases, including ulcerative colitis and Crohn’s disease, where treatment can improve clinical and endoscopic outcomes and promote mucosal healing [164,166,167]. Thus, intestinal disease provides an important example in which therapeutic manipulation of JAK-STAT signaling has progressed from mechanistic studies toward clinical application, although the beneficial effects of pathway inhibition reflect suppression of pathological inflammation rather than direct stimulation of epithelial regeneration.

JAK-STAT signaling also contributes to liver regeneration and neural repair. These examples primarily reflect responses of differentiated cells and progenitors rather than direct evidence of bona fide tissue stem cells. During hepatic injury, IL-6 activates JAK-STAT3 signaling in hepatocytes, promoting survival and cell-cycle entry during the early regenerative response [168,169,170,171]. Experimental disruption of STAT3 impairs hepatocyte proliferation, supporting its importance in physiological liver regeneration. In the nervous system, STAT3 contributes to neuronal survival and axonal regeneration after peripheral nerve injury, while STAT3 activity in Schwann cells supports the repair phenotype and production of neurotrophic factors [172,173]. These findings provide a rationale for therapeutic modulation of JAK-STAT signaling in liver injury and neural repair, although clinical translation remains less advanced than in oncology and inflammatory disease.

However, therapeutic manipulation of JAK-STAT signaling must be approached cautiously. Because the pathway regulates diverse physiological processes, including immunity, hematopoiesis, and tissue regeneration, systemic inhibition can lead to immunosuppression, increased infection risk, and hematologic toxicities [174,175]. Long-term inhibition may also disrupt normal stem cell function and tissue homeostasis, underscoring the importance of dosage, timing, and tissue specificity in therapeutic design.

Advances in molecular biology and drug development are enabling increasingly precise interventions within the JAK-STAT pathway. Emerging strategies include selective STAT inhibitors, modulation of upstream cytokine signaling, and targeting of pathway regulators such as SOCS proteins [176,177]. In addition, novel approaches aimed at disrupting STAT3 dimerization, DNA binding, or mitochondrial functions highlight the growing interest in targeting both canonical and noncanonical STAT activities [178,179]. These approaches may ultimately allow therapeutic manipulation of stem cell signaling networks to promote regeneration while limiting oncogenic risk.

## 9. Emerging Technologies and Systems-Level Approaches

Advances in high-throughput and systems-level technologies are providing new insights into the regulation of JAK-STAT signaling in stem cell biology. Modern experimental approaches allow investigators to examine signaling dynamics, transcriptional networks, and cellular heterogeneity with unprecedented resolution. These tools are revealing how cytokine signaling pathways operate within complex stem cell populations and their surrounding microenvironments [180,181].

Single-cell transcriptomics has become a particularly powerful method for dissecting signaling heterogeneity within stem cell compartments. By profiling gene expression in thousands of individual cells, single-cell RNA sequencing can identify distinct stem and progenitor cell states and reveal transcriptional programs associated with STAT activation. These studies have demonstrated that JAK-STAT signaling is often dynamically regulated across stem cell populations, generating functional heterogeneity that contributes to tissue homeostasis and regenerative capacity [182]. Integration with single-cell ATAC-seq and multi-omics approaches further enables the linking of STAT-dependent transcriptional outputs with chromatin accessibility and regulatory element activity [183,184].

Spatial transcriptomics and imaging-based approaches are further enhancing understanding of how signaling pathways operate within stem cell niches. These technologies allow researchers to map cytokine expression and STAT activation within intact tissues, providing insight into the spatial organization of signaling networks that regulate stem cell behavior. Such analyses are particularly valuable for understanding how local microenvironments shape signaling gradients and influence stem cell fate decisions [185,186]. Emerging multiplexed imaging and in situ sequencing methods now enable simultaneous visualization of signaling components, transcriptional states, and cell–cell interactions at near-single-cell resolution [187].

Computational modeling and systems biology approaches are also contributing to the study of JAK-STAT signaling. Mathematical models of cytokine signaling networks can simulate pathway dynamics, predict feedback regulation, and identify key control nodes that determine signaling outcomes. These approaches have helped explain how differences in signaling thresholds and feedback loops can produce distinct cellular responses to similar cytokine stimuli [188,189]. Work integrating computational simulation with stem cell biology further demonstrates how distinct JAK-STAT signaling dynamics in somatic versus germline stem cells can generate cell-type-specific regulatory outcomes [40,128]. Integration of computational modeling with experimental data is increasingly important for understanding the complex regulatory networks governing stem cell behavior.

Together, these emerging technologies are transforming the study of cytokine signaling in stem cell systems. By combining single-cell analysis, spatial mapping, and computational modeling, researchers are beginning to construct comprehensive frameworks describing how JAK-STAT signaling regulates stem cell function within complex tissue environments [121,190]. Increasingly, these integrative approaches also enable the identification of therapeutic targets and predictive biomarkers by linking signaling dynamics to functional stem cell states.

## 10. Future Directions and Outstanding Questions

Despite substantial progress in understanding the roles of JAK-STAT signaling in stem cell biology, many important questions remain. One major challenge is to determine how distinct STAT family members generate context-specific transcriptional outputs in different stem cell populations. Although STAT proteins share structural similarities and can recognize related DNA motifs, they often regulate distinct gene networks that control stem cell fate decisions. Understanding how cofactors, chromatin context, and signaling crosstalk shape these transcriptional programs will be essential for elucidating the mechanisms underlying pathway specificity.

Another important area for future research involves the integration of JAK-STAT signaling with other regulatory pathways that control stem cell behavior. Stem cells respond to multiple environmental signals simultaneously, including cues from pathways such as Wnt, Notch, Hedgehog, and MAPK. Interactions between these pathways can produce synergistic or antagonistic effects that influence stem cell maintenance and differentiation. Dissecting these complex signaling networks will require both experimental and computational approaches capable of analyzing pathway interactions at multiple levels.

The noncanonical functions of STAT proteins also remain an active area of investigation. Increasing evidence indicates that STAT proteins can regulate mitochondrial activity, chromatin organization, and other cellular processes independently of classical transcriptional signaling. How these noncanonical roles contribute to stem cell maintenance and differentiation is not yet fully understood. The *Drosophila* germline stem cell system, with its well-defined niche and genetic tractability, offers a powerful model to dissect these noncanonical STAT functions in vivo, particularly how unphosphorylated STAT contributes to chromatin organization and stem cell identity across generations.

Finally, a major goal of future research is to translate mechanistic insights into therapeutic strategies. Targeting JAK-STAT signaling holds promise for treating diseases involving stem cell dysregulation, including cancers driven by malignant stem cells and degenerative disorders requiring enhanced regenerative capacity. However, because this pathway regulates diverse physiological processes, therapeutic interventions must be carefully designed to avoid disrupting normal tissue homeostasis.

Addressing these challenges will require interdisciplinary approaches integrating molecular biology, genomics, computational modeling, and translational research. Continued investigation of JAK-STAT signaling in stem cell systems will therefore provide important insights into both fundamental biology and clinical applications. A particularly important frontier is the translation of mechanistic insights from model systems to human disease. Germline and somatic mutations in JAK-STAT pathway components cause a spectrum of human disorders, ranging from inborn errors of immunity to myeloproliferative neoplasms and leukemias. Understanding how specific mutations alter STAT activity thresholds in distinct stem and progenitor cell populations will be essential for developing genotype-directed therapies that restore normal signaling without disrupting essential physiological functions.

## 11. Conclusions

The JAK-STAT signaling pathway represents a central regulatory mechanism linking extracellular cytokine signals to transcriptional programs that control stem cell behavior. Across diverse biological systems, this pathway regulates key processes, including stem cell self-renewal, proliferation, quiescence, and differentiation. In pluripotent stem cells, STAT signaling contributes to maintenance of the undifferentiated state, whereas in adult tissues, the pathway integrates cytokine signals from the microenvironment to coordinate regeneration and tissue homeostasis.

Recent studies have revealed that JAK-STAT signaling operates within a broader regulatory framework involving stem cell niches, metabolic pathways, and epigenetic mechanisms. These interactions provide multiple levels at which JAK-STAT activity can be therapeutically modulated, including cytokine receptor signaling, JAK kinase activity, STAT3/STAT5 function, and noncanonical STAT regulation of metabolism and chromatin. At the same time, dysregulation of JAK-STAT signaling contributes to pathological conditions, including inflammatory diseases and cancers characterized by stem-like cell populations.

Advances in single-cell technologies, spatial transcriptomics, and computational modeling are providing new insights into how JAK-STAT signaling operates within complex stem cell systems. These approaches are revealing previously unrecognized heterogeneity in signaling states and uncovering regulatory mechanisms that govern stem cell function at the systems level. Such approaches may help identify tissue-specific therapeutic targets and biomarkers that enable more selective modulation of JAK-STAT signaling.

Continued investigation of JAK-STAT signaling in stem cell biology will deepen understanding of how extracellular signals shape cellular identity and tissue regeneration. Clinically, the challenge is to distinguish beneficial, transient JAK-STAT activation that supports tissue repair from persistent signaling that promotes inflammation, stem cell dysfunction, or malignant transformation. Therapeutic strategies that selectively inhibit pathological JAK-STAT activity or modulate specific STAT functions in a tissue- and context-dependent manner may therefore provide opportunities in both regenerative medicine and oncology.

## Figures and Tables

**Table 1:** Representative therapeutic strategies targeting the JAK-STAT pathway.

Therapeutic Strategy	Representative Agents/Approaches	Principal Target	Clinical Status/Application	Relevance to Stem Cell Biology
**JAK1/2 inhibition**	**Ruxolitinib**	JAK1/JAK2	Clinically established; myelofibrosis and other hematologic disorders	Suppresses aberrant cytokine signaling; potential modulation of pathological stem/progenitor-cell activity
**JAK1/3 inhibition**	**Tofacitinib**	JAK1/JAK3	Clinically established; inflammatory and autoimmune diseases	Modulates cytokine-dependent stem-cell and tissue inflammatory environments
**JAK1/2 inhibition**	**Baricitinib**	JAK1/JAK2	Clinically established; inflammatory diseases	Alters cytokine signaling that influences tissue repair and immune–stem-cell interactions
**Selective JAK2 inhibition**	**Fedratinib**	JAK2	Clinically established; myelofibrosis	Targets pathological JAK2 signaling in hematopoietic disease
**Selective JAK1 inhibition**	**Upadacitinib**	JAK1	Clinically established; inflammatory diseases	Modulates cytokine signaling relevant to tissue inflammation and regeneration
**STAT3-directed approaches**	STAT3 inhibitors, antisense oligonucleotides, or other STAT3-modulating strategies	STAT3	Primarily experimental/preclinical; selected clinical investigation	Potentially targets STAT3-dependent stemness, survival, metabolism, or tumor-promoting signaling
**STAT5-directed approaches**	STAT5 inhibitors/modulators	STAT5	Experimental/preclinical	May interfere with STAT5-dependent maintenance of normal or malignant stem/progenitor populations
**Cytokine receptor modulation**	Targeting IL-6/IL-6R, LIF/LIFR, or related cytokine pathways	Upstream JAK-STAT activation	Clinically established for selected inflammatory diseases; broader applications under investigation	May alter niche-derived signals controlling stem-cell proliferation, differentiation, and regeneration
**Noncanonical STAT targeting**	Modulation of mitochondrial STAT3 or STAT–chromatin/HP1 interactions	Noncanonical STAT functions	Experimental/preclinical	Emerging strategy targeting metabolic or epigenetic mechanisms associated with stem-cell and cancer-cell states

Clinical status refers to established therapeutic use of representative agents; emerging STAT-directed and noncanonical STAT approaches remain primarily investigational. Therapeutic effects on bona fide normal stem cells, progenitors, or cancer stem cells may differ according to tissue, disease context, and signaling state.

## Data Availability

No new data were created or analyzed in this study. Data sharing is not applicable to this article.
